# The Nature of Nanodisc Lipids Influences Fragment‐Based Drug Discovery Results

**DOI:** 10.1111/cbdd.70080

**Published:** 2025-03-14

**Authors:** Tim G. J. Knetsch, Henri van Son, Masakazu Kobayashi, Marcellus Ubbink

**Affiliations:** ^1^ Leiden Institute of Chemistry Leiden University Leiden the Netherlands; ^2^ ZoBio B.V. Leiden the Netherlands

**Keywords:** cytochrome P450, drug‐membrane interactions, fragment‐based drug discovery, nanodiscs, surface plasmon resonance

## Abstract

Membrane proteins (MPs) are important yet challenging targets for drug discovery. MPs can be reconstituted in protein‐lipid Nanodiscs (NDs), which resemble the native membrane environment. Drug‐membrane interactions can affect the apparent binding stoichiometry and affinity, as well as the kinetics of ligands for a particular target, which is important for the extrapolation to pharmacokinetic studies. To investigate the role of the membrane, we have applied fragment‐based drug discovery (FBDD) methods to cytochrome P450 3A4 (CYP3A4), reconstituted in NDs composed of different phosphocholine lipids: 1‐palmitoyl‐2‐oleoyl‐sn‐glycero‐3‐phosphocholine (POPC), 1,2‐dimyristoyl‐sn‐glycero‐3‐phosphocholine (DMPC), dipalmitoylphosphatidylcholine (DPPC), or 1,2‐diphytanoyl‐sn‐glycero‐3‐phosphocholine (DPhPC). Surface plasmon resonance screening of fragments and marketed drugs revealed extensive binding to the empty ND, correlating with analyte hydrophobicity, and the binding was critically dependent on ND lipid composition. POPC NDs showed much higher binding of fragments than DMPC and DPhPC NDs, resulting in a lower hit rate for CYP3A4 in POPC NDs, which demonstrated that the choice of the ND lipid is crucial to the outcome of a screen. The number of binders that were rejected based on atypical binding kinetics was lower for monomeric CYP3A4 in NDs than for non‐native oligomeric CYP3A4 without the ND. Several fragments were exclusively identified as hits for CYP3A4 in the presence of the ND membrane. It is concluded that the nature of the ND is a critical factor for fragment screening of membrane proteins.

## Graphical abstract

Fragment‐based drug discovery (FBDD) was applied to cytochrome P450 3A4 reconstituted in Nanodiscs (NDs) with various lipid compositions. The choice of ND lipid influenced drug –membrane interactions and fragment hit rates, demonstrating the critical role of the membrane environment in fragment screening for membrane proteins.

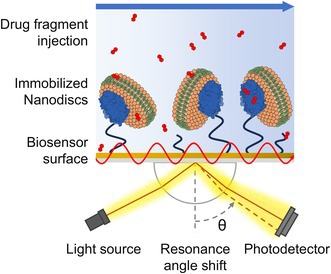

Fragment‐based drug discovery (FBDD) has emerged as a strategy complementary to high‐throughput screening in the identification of molecules for hit‐to‐lead campaigns of difficult targets (Erlanson 2011; Bon et al. 2022). A significant advantage of FBDD over traditional high‐throughput screening is that the chemical space is sampled more efficiently using a smaller library, resulting in hits that possess a higher average binding energy per atom (Hopkins et al. 2004). Small drug fragments (~100–300 Da) serve as starting points for expansion or assembly into higher affinity lead compounds, by maintaining chemical ‘room’ for improvement. Fragments can be expanded or ‘grown’ by structure‐based rational design, by combining structural elements of multiple fragment hits, or by merging them with known binders for a particular site. Fragment libraries generally comply with the so‐called ‘rule of three’ (Ro3) (Congreve et al. 2003), which was adapted from Lipinski's rule of five (Lipinski et al. 2012). The Ro3 states that fragments should have a Mw ≤ 300 Da, hydrogen bond donors (HBD) ≤ 3, hydrogen bond acceptors (HBA) ≤ 3 and the computed logarithm of the octanol–water partition or distribution coefficient (cLogP/cLogD) ≤ 3. ClogP refers to the computed concentration ratio of the un‐ionized compound, while clogD includes both ionized and un‐ionized forms at a given pH (Bhal et al. 2007). Due to the small size of fragments, binding affinities are relatively low with equilibrium dissociation constants (*K*_D_) in the μM–mM range. Thus, to obtain high quality binding data, sensitive analytical techniques are required. Only a few techniques can accurately measure protein‐fragment binding, the most prevalent of which are x‐ray crystallography, nuclear magnetic resonance (NMR) spectroscopy, thermal shift assays (TSA) such as differential fluorimetry (DSF), and surface plasmon resonance (SPR) (Bon et al. 2022).

Membrane proteins (MPs) make up a significant portion of the human proteome (22%–30%) (Overington and Hopkins 2006; Fagerberg et al. 2010), and a study from 2006 revealed that around 60% of the therapeutic drugs approved at that time targeted molecules located on the cell surface (Overington and Hopkins 2006). Important pharmaceutical targets include ion channels, transporters, G protein‐coupled receptors (GPCRs), and cytochromes P450 (CYPs), which play crucial roles in drug metabolism (Bakheet and Doig 2009; Patching 2014). Additionally, for many soluble targets, therapeutics must first cross the cell membrane to access the intracellular space, underscoring the importance of interactions between medicine and membrane. Drug screening of MPs poses significant challenges because expression levels are typically low and the necessity for solubilization and purification in detergents can compromise MP activity and stability. Furthermore, the presence of excess detergent in the screening buffer can result in the sequestering of compounds inside micelles (Congreve et al. 2011; Früh et al. 2010). Nevertheless, successful screening campaigns of small molecules and fragments have been conducted with detergent‐solubilized MPs, using techniques such as SPR (Congreve et al. 2011; Rich et al. 2011; Christopher et al. 2013; Huber et al. 2017; Shepherd et al. 2014), and target‐immobilized NMR screening (TINS) (Früh et al. 2010; Siegal and Hollander 2009; Chen et al. 2012), which require modest quantities of MP. An appealing alternative to detergent solubilization is the reconstitution of MPs in model membranes, such as protein‐lipid Nanodiscs (NDs), because this system better resembles the native environment of cellular membranes (Denisov et al. 2004; Simonsen 2016). NDs also better mimic small molecule partitioning to the membrane, which can affect the apparent affinity and stoichiometry of analyte binding for the target MP (Nath et al. 2007). Drug‐membrane interactions could be especially important when translating in vitro binding or kinetic data to in vitro pharmacokinetics. NDs have been used effectively for the stable immobilization of MPs for SPR (Pearson et al. 2006; Bocquet et al. 2015; Das et al. 2009) and TINS (Früh et al. 2010) screening experiments, and they are amenable to a wide variety of surface chemistries for immobilization, using either the lipid or protein components (Trahey and Atkins 2015). NDs are homogeneous in size, provide accessibility from both sides of the membrane, and allow precise control of protein and lipid composition. In spite of these advantages, only a handful of fragment screens against ND‐incorporated MPs have been conducted (Früh et al. 2010; Fujimoto et al. 2021), and the role of ND lipid composition in fragment binding has not been investigated thoroughly.

To address this knowledge gap, a prototypical membrane protein, cytochrome P450 (CYP) 3A4 (CYP3A4), was selected and assembled into NDs comprising different lipids. The main aim of the study is to assess the impact of ND lipid composition on small molecule and fragment binding to both ‘empty’ and CYP3A4‐incorporated NDs. SPR was chosen as the detection method because it requires minimal sample and allows for label‐free, highly sensitive, and automated screening of multiple NDs.

CYPs play a crucial role in the biotransformation of xenobiotics in the human body, including 70%–80% of marketed drugs (Zanger and Schwab 2013), numerous endogenous ligands, and other foreign natural products. CYP3A4 is the most prevalent human isoform (Guengerich 2008; Šrejber et al. 2018), and provides a comprehensive model MP because it naturally binds diverse substrates that can access the enzyme from the aqueous solution and lipid bilayer (Šrejber et al. 2018; Berka et al. 2013; Monk et al. 2014). Consequently, fragments with varying hydrophobicity can potentially bind to CYP3A4, providing a holistic view of library binding to a MP inside a ND model membrane. CYP3A4 has a single helix transmembrane domain (TMD) that is likely positioned near the edge of the ND for the majority of the time, as was shown by small‐angle x‐ray scattering and MD simulations (Skar‐Gislinge et al. 2015). The membrane is crucially important for trafficking hydrophobic substrates to the heme‐containing active site from the distal face of CYP3A4 (with respect to the TMD), which is partially immersed inside the membrane (Monk et al. 2014; Lonsdale et al. 2014) (Figure 1).

**FIGURE 1:**
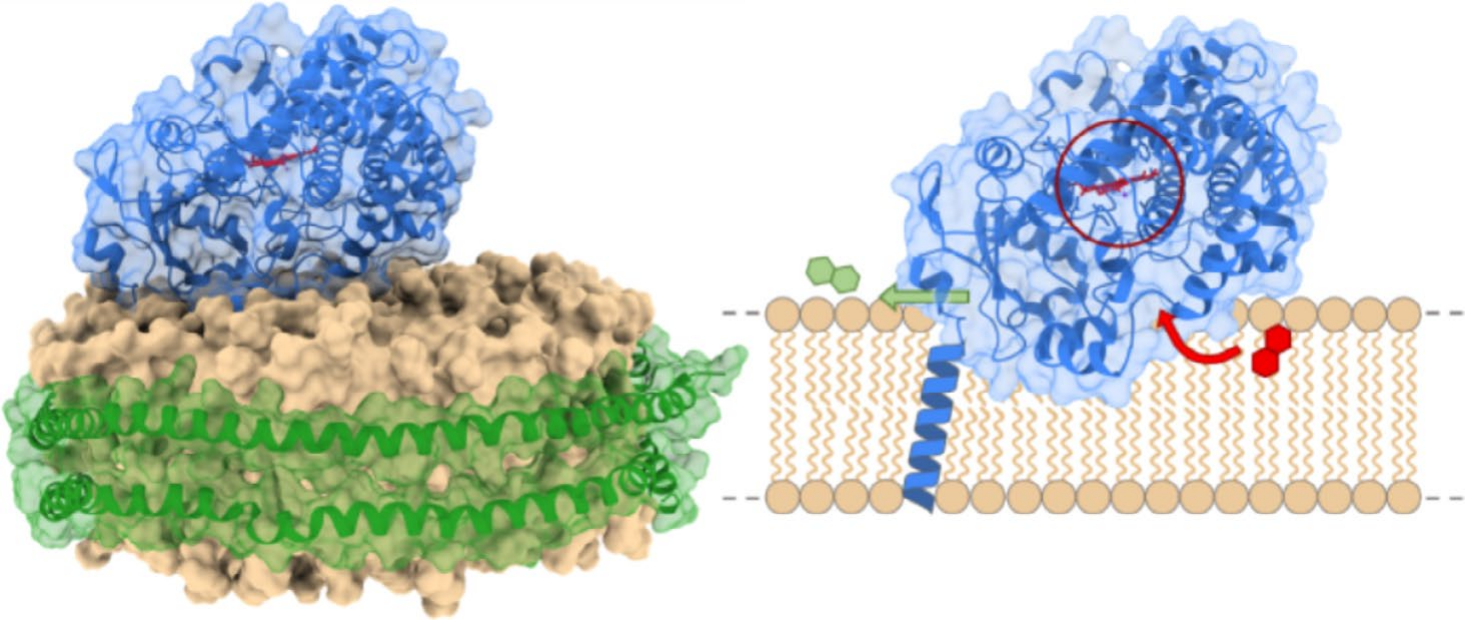
A model of CYP3A4 in a ND. The crystal structure of CYP3A4 (PDB: 1TQN) (Marcink et al. 2019) is shown partially immersed in the ND membrane. The ND model (PDB: 6CLZ) (Yano et al. 2004) was constructed with CHARMM GUI (Jo et al. 2008). Amino acid residues 1–27 of CYP3A4, including the membrane domain are not present in the crystal structure, therefore the transmembrane alpha helix is shown as a cartoon. Hydrophobic substrates (red hexagons) access the heme‐containing active site (encircled in red) through various access channels in contact with the membrane and the more hydrophilic products (green hexagons) can leave the enzyme through an egress channel near the phospholipid headgroup region, at the interface with the aqueous environment. ChimeraX was used to render the models of CYP3A4 and the ND (Meng et al. 2023).

In this study, we compare NDs comprising a single type of lipid, featuring neutral, zwitterionic phosphatidylcholine headgroups, and varying acyl chains (Figure 2). These include: 1‐palmitoyl‐2‐oleoyl‐sn‐glycero‐3‐phosphocholine (POPC; 16:0–18:1, *T*_m_ = −2°C), 1,2‐dimyristoyl‐sn‐glycero‐3‐phosphocholine (DMPC; 14:0, *T*_m_ = 24°C), dipalmitoylphosphatidylcholine (DPPC; 16:0, *T*_m_ = 41°C), and 1,2‐diphytanoyl‐sn‐glycero‐3‐phosphocholine (DPhPC; 16:0–4ME, exhibiting no defined *T*_m_ for −120°C to 120°C). Considerable binding of lipophilic small molecules and fragments to the ND membrane was observed, correlating strongly with analyte cLogP/D, and critically depended on the nature of the lipid. DMPC and DPhPC NDs provided better referencing systems for SPR measurements of incorporated CYP3A4 than POPC NDs did.

**FIGURE 2:**
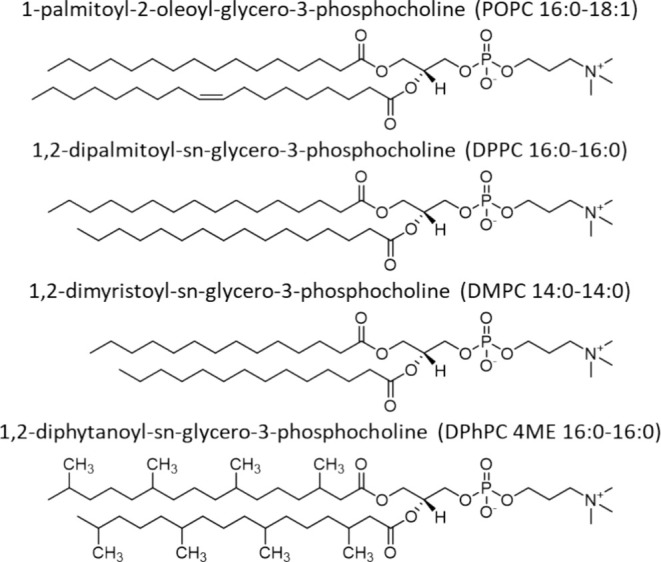
Chemical structures of the phospholipids used for ND assembly.

## 1 Experimental Section

### 1.1 Nanodisc Assembly Procedures

MSP1D1 was expressed and purified as previously described (Bayburt et al. 2002; Knetsch and Ubbink 2024a). Chemically synthesized lipids were bought from Avanti polar lipids. Lipid films and empty NDs were prepared as previously described (Knetsch and Ubbink 2024a). CYP3A4 containing a 6× histidine purification tag was recombinantly expressed as previously described (Knetsch and Ubbink 2024b; Denisov et al. 2006). The concentration and integrity of CYP3A4 were determined (Fe^2+^ CO/Fe^2+^ difference spectra) according to the method of Omura and Sato (Omura and Sato 1964). CYP3A4 NDs were prepared using MSP1D1 without the his‐tag, which was cleaved off using TEV protease as described previously (Knetsch and Ubbink 2024b).

### 1.2 Sensor Chip Surface Preparations

Biosensor experiments were performed on a Biacore 1S+ (Cytiva, Uppsala, Sweden) using a poly‐Ni^2+^‐NTA, NiHC1000M chip (Xantec). The immobilized targets were diluted to 2 μM in capture buffer and captured by Ni^2+^‐affinity using the his‐tag on MSP1D1 for empty NDs and the his‐tag on CYP3A4 for the other samples. CYP3A4 without ND was captured to ~4000 RU. Empty and CYP3A4 NDs were captured to 4000–6500 RU.

### 1.3 SPR Analysis of Tool Compounds

Known binders of CYP3A4 (tool compounds) were found using the online Drugbank database: Cytochrome P450 CYP3A4 Substrates, accession number DBCAT003919, and are summarized in Table S1. The SPR system was equilibrated in 0.1 M potassium phosphate (KPi), pH 7.4, 0.15 M NaCl, 10 μM EDTA, 5% Glycerol, and 1% DMSO (running buffer). Tool compounds were injected for 60 s at a concentration of 1 or 10 μM at a flowrate of 30 μL/min. Sensorgrams were reference subtracted using the signal from the reference channel in the Biacore Insight Evaluation Software (Cytiva). A five‐point solvent correction was performed with 0.7%–1.8% (v/v) DMSO to compensate for DMSO mismatch between running buffer and analyte samples. The data were corrected by the subtraction of blank injections before and after each compound injection series.

### 1.4 SPR Fragment Screening

A total of 140 Fragments representative of the ZoBio screening library were injected at 250 and 500 μM, with a 20 s contact time and 40 s dissociation. The same running buffer was used as for the tool compounds (previous section). One percent DMSO blank injections were performed in between every five fragment injections to correct for the response from the injection buffer. The injection needle was washed with 50% DMSO before and after every five fragments and between blank injections to remove any residual fragments.

### 1.5 Analysis of Fragment Binding and Hit Selection Criteria

Fragments showing occupancy below 0% or significant interaction with the reference surface were defined as reference binders. Fragments with an occupancy < 50% were classified as non‐binders. Fragments with an occupancy between 50% and 300%, displaying fragment‐like ‘squared’ sensorgrams were classified as hits. Fragments were flagged when they showed non‐fragment‐like sensorgrams, for example, due to atypical dissociation or having a positive slope during association. Flagged binders were rejected due to a high likelihood of being affected by aspecific interactions and were not counted as hits. Fragments with an occupancy > 300% were classified as super‐stoichiometric binders and were not counted as hits.

## 2 Results

### 2.1 Lipid Composition Affects the Binding of Hydrophobic Analytes to the Nanodisc Model Membrane

**FIGURE 3:**
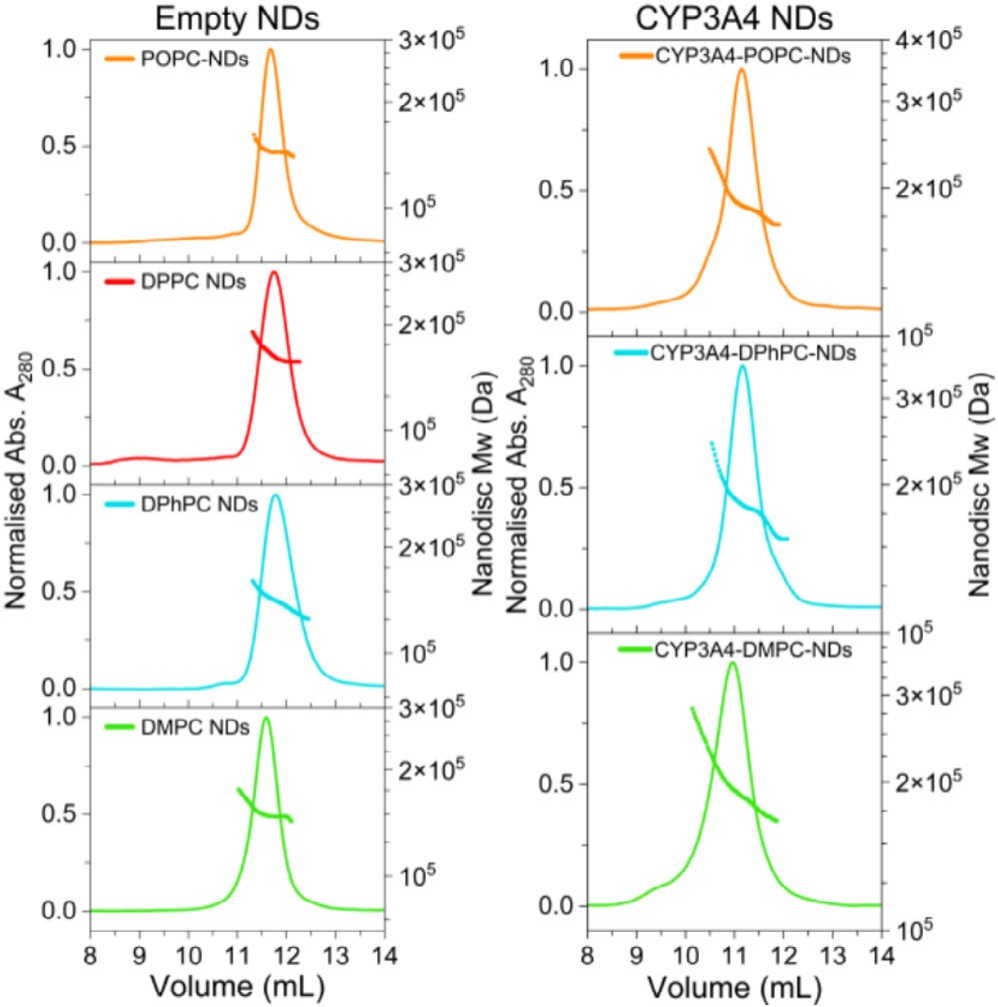
Empty‐ (left) and CYP3A4‐NDs (right) comprising POPC (orange), DPPC (red), DPhPC (cyan) and DMPC (green) lipids, analyzed by SEC‐MALS. The total ND Mw is shown as a dotted line (right axis) overlaying the normalized 280 nm absorbance curve (left axis).

**FIGURE 4:**
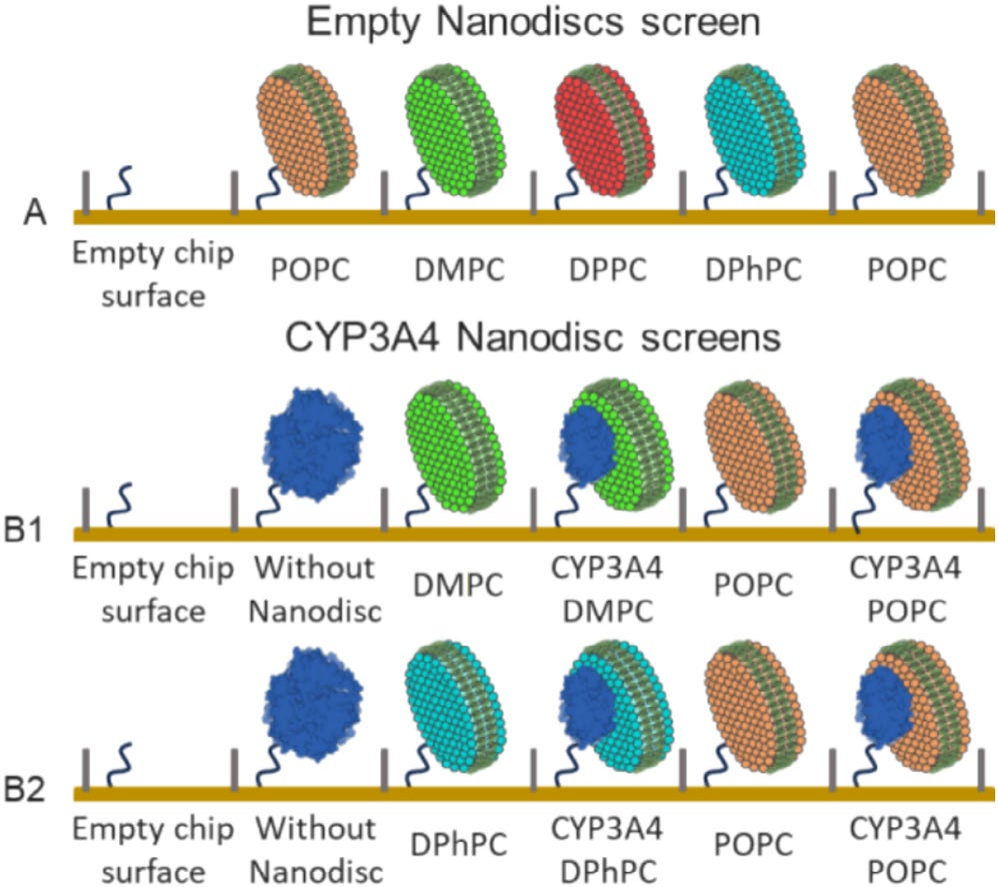
SPR surface representation for empty NDs (A) and CYP3A4 inside and without ND (B1, B2). CYP3A4 without ND is presented as a non‐native oligomer. The empty chip surface was used as a reference for the empty NDs and CYP3A4 without ND. For the CYP3A4‐NDs, the empty NDs containing the same lipids were used as a reference. The CYP3A4‐NDs were screened in two consecutive runs; CYP3A4 without ND and CYP3A4‐POPC‐NDs were included in both screens B1 and B2.

**FIGURE 5:**
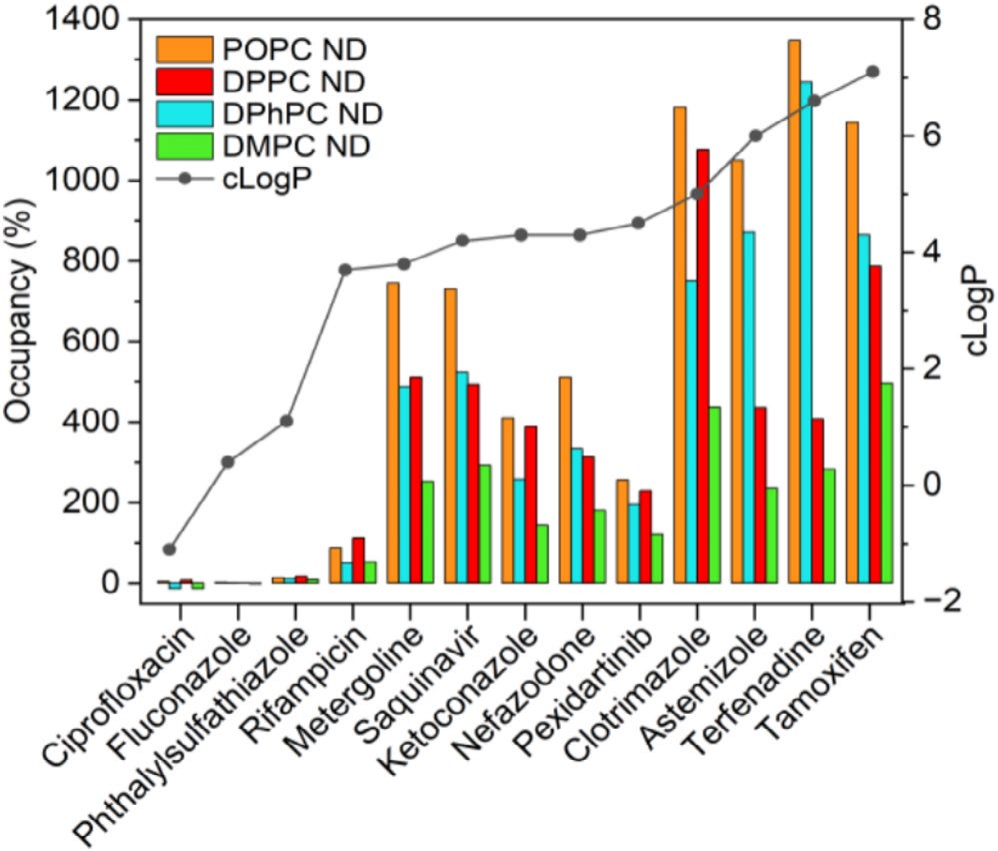
Binding of tool compounds (10 μM) to empty NDs comprising POPC, DPPC, DPhPC, and DMPC lipids. Binding of lipophilic analytes (cLogP > 1.1) was highest for POPC>DPPC>DPhPC>DMPC NDs. Hydrophilic analytes (cLogP < 1.1) show no binding to empty NDs. The line connecting the cLogP values is drawn to guide the eye.

Empty and CYP3A4 NDs containing POPC, DPhPC, or DMPC lipids were assembled and purified by size‐exclusion chromatography (SEC). DPPC membranes have a *T*_m_ of 41°C, which is detrimental for the incorporation of CYP3A4 and, therefore, this lipid was only used to assemble empty NDs. ND sizes were characterized by SEC coupled to multi‐angle light scattering (SEC‐MALS) (Figure 3). Detailed characterization of ND composition and thermal stability was done in a previous study for empty NDs (Knetsch and Ubbink 2024a) and CYP3A4 NDs (Knetsch and Ubbink 2024b). First, the binding of marketed drugs (tool compounds) to empty NDs was measured to evaluate the degree of binding to the membrane model systems used afterward for screening of CYP3A4 NDs with fragments. The empty NDs were captured by nickel affinity on a poly‐Ni^2+^‐NTA sensor chip, and the surface immobilization pattern in the six channels arranged in series on the chip is shown in Figure 4A. Thirteen tool compounds known to bind to CYP3A4 and with a broad range of cLogP values were injected and exposed to the immobilized NDs. To compare the binding of analytes to NDs of different sizes, the binding was defined as “percent occupancy” which is calculated from the blank corrected SPR response units (RU), after reference subtraction, and normalized for the Mw of the ND and analyte (see Supporting Information for details). An occupancy of 100% indicates that, on average, one analyte molecule was bound per ND. The analytes with high cLogP values (> 3.5) bound to all empty NDs, with a clear disparity in the degree of binding observed for the different lipids (Figure 5). POPC NDs consistently displayed the highest small molecule binding, followed by DPhPC and DPPC NDs. DMPC NDs always showed the lowest binding, 40 to 80% less than POPC NDs, depending on the analyte. In some instances, more than 10 drug molecules were bound per ND at only 10 μM concentration. The three most hydrophilic analytes (cLogP < 1.1) did not show significant binding to any of the empty NDs at 10 μM.

Next, we attempted to detect the binding of the tool compounds to CYP3A4 in NDs (Figure 4B1). Given the extensive binding to the empty NDs, a concentration of only 1 μM was used in this experiment to limit ND binding as much as possible (Figure S1). Among the selected compounds were high‐affinity inhibitors of CYP3A4, such as the azole antifungals (Pearson et al. 2006), so CYP3A4 binding was expected to be detectable at this concentration. However, for most drugs, binding to CYP3A4 was not detected at 1 μM (occupancy < 30%, ≲ 4 RU). Since binding to CYP3A4 was mostly obscured by the molecules' affinity for the ND membrane model, we proceeded with the less hydrophobic fragment library.

### 2.2 Fragment Binding to Empty NDs Correlates Strongly With Analyte cLogP

A pilot library of 140 fragments, representative of the ~2000 fragment ZoBio library (Siegal et al. 2007) was used to screen against NDs with and without CYP3A4. Average physicochemical properties of the entire ZoBio fragment library are summarized in Table S2. A fresh surface was prepared using the empty NDs, as illustrated in Figure 4A, and each fragment was injected at two concentrations, 250 and 500 μM. Fragment binding trends were comparable for both concentrations tested, with 500 μM displaying ~1.7‐fold higher binding than 250 μM. Data collected at 500 μM will be discussed here, and the data for the 250 μM fragment injections can be found in the Supporting Information (Figure S2 & S3).

**FIGURE 6:**
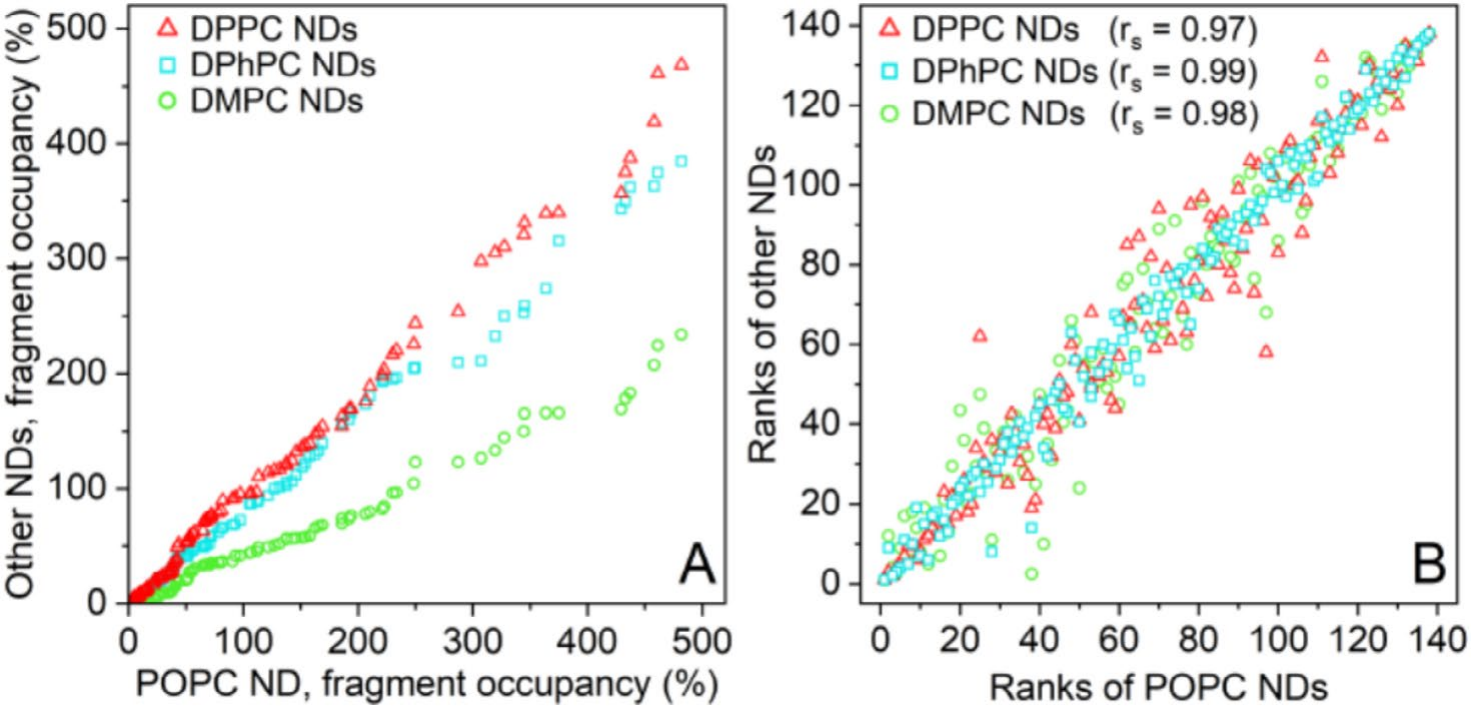
Fragment binding occupancy (%) rank comparison. (A) Fragment occupancy to DPPC, DPhPC and DMPC NDs was plotted against those for POPC ND. In each case the data are ordered by increasing occupancy, so the symbols do not represent the same fragment for the horizontal and vertical axes. The plot shows that fragment binding to the DMPC NDs was lower than the other types of NDs. (B) The rank number of the fragment binding to DPPC, DPhPC and DMPC NDs was plotted against the rank for the same fragment binding to POPC NDs. The higher the correlation is, the more similar the fragment binding behavior of the POPC ND and the other ND type, *r*_s_ = 0.99, 0.98 and 0.97 for DPhPC, DMPC and DPPC NDs, respectively.

**FIGURE 7:**
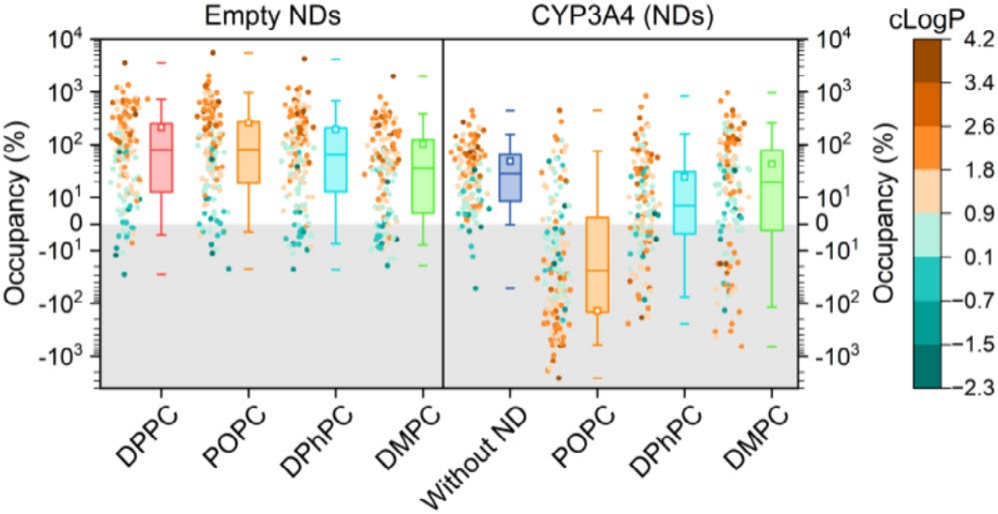
Fragment binding (occupancy, %) at 500 μM to empty NDs, CYP3A4 without ND, and CYP3A4 NDs. The boxes indicate the interquartile range (middle 50%), the median (line within the box), the average (open squares), the whiskers cover 5%–95% of the data, and the outermost data points are indicated by the horizontal dashes. Binding data (dots) were colored according to fragment cLogP (scale on the right). Note that for CYP3A4‐POPC‐NDs, the binding to the reference (empty ND) is noticeably higher, resulting in a negative occupancy for the majority of the hydrophobic fragments. The responses for CYP3A4 NDs were normalized using fluconazole control injections (method section).

The fragment binding response was ranked from lowest to highest, and the ranks were plotted for DPPC, DPhPC, and DMPC NDs against the POPC NDs (Figure 6). Similar to the drug molecules, fragment binding was highest for the POPC NDs, followed by DPPC and DPhPC NDs. Interestingly, fragment binding to the DMPC NDs was considerably lower than for the other types of NDs. It was expected that at the screening temperature of 10°C, the two saturated lipids, DMPC (14:0) and DPPC (16:0), would be most comparable and would show decreased fragment binding due to tight packing in the gel phase, compared to the POPC and DPhPC, which are in the liquid crystalline phase. However, under these conditions, DMPC NDs show markedly lower fragment binding than DPPC NDs, suggesting the lipid phase was not the cause of this effect. Alternatively, a lower fragment occupancy could be explained by the shorter acyl chain length of DMPC (Drabik et al. 2020), resulting in a hydrophobic core that is smaller than for the other lipids. Spearman's correlation coefficients (*r*_s_), which can range from −1 (inverse correlation), via 0 (not correlated) to 1 (positive correlation) (Schober and Schwarte 2018), were calculated for the ranks of fragment binding to the different types of NDs compared to the ranks for binding to POPC NDs (Figure 6B). A strong positive correlation was found for all types of NDs (*r*_s_ > 0.97), indicating that the degree of binding is mostly determined by the nature of the fragment and less by the type of lipid in the NDs. In particular, POPC and DPhPC NDs show very similar fragment ranking. The cLogP/D was a good predictor of ND affinity (*r*_s_ = 0.63–0.68, for all NDs). This suggests that the hydrophobic effect drives the partitioning of fragments toward the ND membrane environment. Under the experimental conditions, cLogD (pH = 7.4) was not a better predictor of fragment partitioning inside empty NDs than cLogP; however, the tested fragments have relatively low Mw, so cLogP and cLogD will be closer than for larger molecules. The fragment binding distributions are summarized in Figure 7. Data points (dots) are colored based on cLogP value. For the empty NDs, the fragments with moderate‐to‐high cLogP (> 1) values were generally clustered above the median, and lower cLogP (< 1) fragments are mostly distributed below the median. The most lipophilic fragment (cLogP = 3.7) displayed a stoichiometry of 20–50 fragments per ND, depending on lipid type, showing that a relatively small ND membrane can accommodate a large number of fragments. For some fragments, the occupancy is negative, meaning that there was more binding to the empty chip surface in the reference channel than to the ND, but this is not very significant (< −20% occupancy). Some fragments with relatively low cLogP displayed binding to the empty NDs, and qualitative inspection suggests these are amphiphilic fragments, resulting in a comparatively low cLogP. Such molecules might orient parallel to the lipids near the headgroup region of the ND membrane. Examples of fragment structures are shown in Table S4.

### 2.3 Fragment Screen of CYP3A4 NDs

Binding to CYP3A4 was measured using the same library of 140 fragments as for the empty NDs. CYP3A4 reconstituted in three types of NDs was immobilized on the SPR chip surface (Figure 4B1 and B2). As a control, the enzyme was also immobilized in the absence of detergent or NDs, in a non‐native oligomeric state, in which the protein is still active (Table S3). Due to leaching of CYP3A4 NDs from the SPR Ni^2+^‐conjugated chip surface, some protein was lost during the 34 h‐long screens. For that reason, fragment responses were normalized using control injections of fluconazole (Figure S4). This compound is a classical type‐II CYP inhibitor that coordinates the heme iron with a 1:1 stoichiometry (Sevrioukova 2019). Fluconazole (cLogP = 0.4) did not show any binding to the empty NDs at 100 μM. SPR absorbance titrations of CYP3A4 in NDs yield a *K*_D_ of ~10–20 μM (Figure S5), in accordance with published data (Sevrioukova 2019; Godamudunage et al. 2018). SPR titrations of fluconazole to immobilized CYP3A4 with and without NDs yielded similar *K*_D_ values as for spectral titrations (Figure S6, and Table S3). Thus, the SPR response of fluconazole is a good indicator of the amount of active CYP3A4 left on the SPR sensor chip.

Fragment binding to CYP3A4 with and without NDs was classified by occupancy; as reference binder (< 0%), no binder (0%–50%), binder (50%–300%) or super‐stoichiometric binder (> 300%), see Table 1. Some of the fragment binders were rejected for atypical behavior, such as slow dissociation or when displaying a slope during association. Because of their small size, it is expected that fragments display fast binding kinetics (high *k*_on_/*k*_off_), and atypical behavior is likely a result of aspecific interactions (FitzGerald et al. 2024).

**TABLE 1:** Summary of the fragment screen (500 μM) for CYP3A4 with and without NDs. Fragments were classified based on binding occupancy (%). The relationships between the hits are shown in a Venn diagram (Figure 8).

Fragments classified as	Occupancy (%)	CYP3A4 without ND^a^	CYP3A4‐POPC‐ND^a^	CYP3A4‐DPhPC‐ND^b^	CYP3A4‐DMPC‐ND^a^
Reference binder^c^	< 0	7	101	51	39
No binder	0–50	84	27	65	58
Binder	50–300	47	11	21	37
Super‐stoichiometric binder	> 300	2	1	3	6
Rejected binder^d^, number (%)		14 (30)	2 (18)	2 (10)	4 (11)
Hits		33	9	19	33

^a^ CYP3A4 sample weres measured only in screen B1.

^b^ DPhPC‐CYP3A4‐NDwase measured only in screen B2.

^c^ Fragments showing occupancy below 0% or significant interaction with the reference surface were defined as reference binders.

^d^ Fragment binders (50%–300%) flagged for atypical behavior, such as slow dissociation from the main or reference channels, were not counted as hits.

A high number of fragments bound to CYP3A4 without the ND, and their occupancy was positively correlated with fragment cLogP (*r*_s_ = 0.63), even in the absence of a membrane environment. However, a large portion of these binders was rejected due to atypical behavior (30%), suggesting that the correlation between cLogP and fragment binding to oligomeric CYP3A4 could be, in part, due to aspecific hydrophobic interactions. A lot of fragments displayed higher binding to the empty ND in the reference channel than the CYP3A4 ND. A negative fragment occupancy for CYP3A4 NDs does not necessarily mean that these fragments are not binding to CYP3A4. For those fragments that display a high binding stoichiometry for the empty ND, any additional binding to CYP3A4 may be obscured by the subtraction of the reference signal. For CYP3A4 in POPC‐NDs, this effect was very pronounced, implying that a POPC ND is less suitable as a referencing system for SPR than DPhPC and DMPC NDs. The high fragment binding to the empty POPC ND reference resulted in the lowest number of hits for CYP3A4‐POPC‐NDs. Fragment binding trends to CYP3A4 in DMPC and DPhPC NDs were comparable (*r*_s_ = 0.78), but the fragment occupancy for CYP3A4 in DPhPC NDs is generally lower than for CYP3A4‐DMPC‐ND. A Mann Whitney test showed a significant difference in median fragment occupancy (P ≈ 0.03) for these two NDs, indicating that fragment binding differed based on the ND membrane type. Most hits for CYP3A4 in NDs were found using DMPC NDs, with only 11% of the binders displaying atypical behavior at 500 μM fragment concentration (0% at 250 μM). This observation suggests that the quality of the measured interactions and the reliability of hits is higher than for CYP3A4 without the ND (Table 1). Also, for CYP3A4‐DPhPC‐NDs, the number of rejected fragment binders was low, 10% and 0% at 500 and 250 μM, respectively.

**FIGURE 8:**
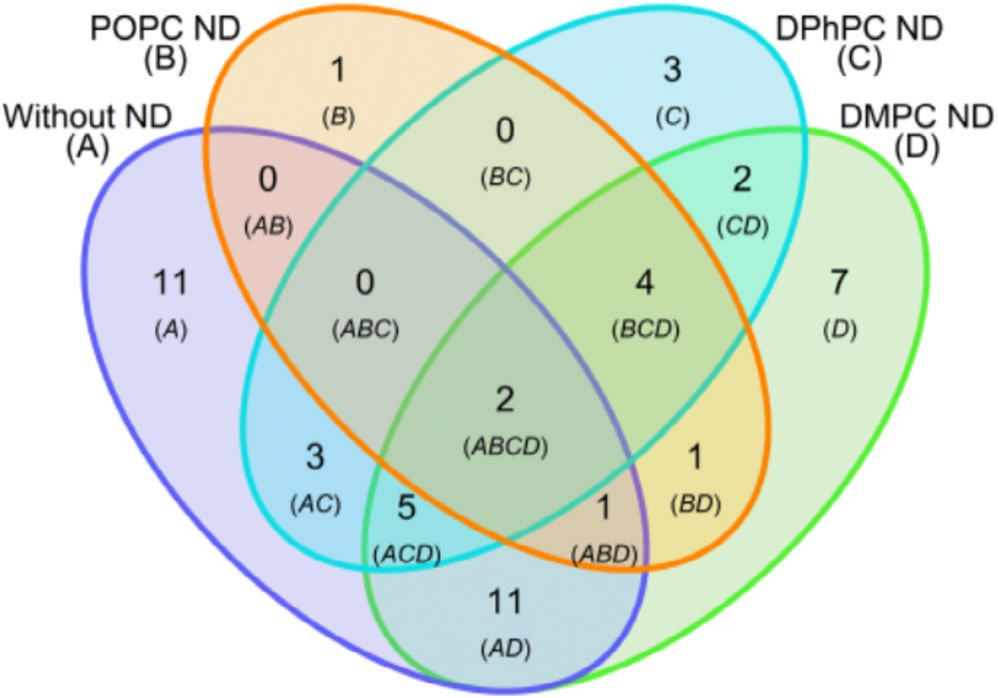
Venn diagram of the fragment hits for CYP3A4 with and without ND. Example fragment hit structures are shown in Table S4.

To probe the degree of similarity, the overlap in hits for different ND types and free CYP3A4 was visualized in a Venn diagram (Figure 8). Reasons why fragments bind differently to CYP3A4 in NDs or free CYP3A4 are the oligomerization state of the enzyme, the differences in the physicochemical environment, and in the substrate access channels of CYP3A4 in the presence of a lipid bilayer (Lonsdale et al. 2014). Out of 33 hits, 11 hits were uniquely found for CYP3A4 without the ND, being hydrophobic fragments (average cLogP of ~2.3) that displayed significant binding for the empty ND membrane with an average occupancy of ~700%, ~550%, ~500%, and ~300% for NDs comprising POPC, DPPC, DPhPC, and DMPC lipids, respectively. The extensive binding to all empty NDs suggests that these fragments interact aspecifically with the hydrophobic region and transmembrane tail of the enzyme. Only one of these 11 hits has a relatively low occupancy for the empty NDs (~50% for DMPC NDs) and was classified as a super‐stoichiometric binder for CYP3A4 in NDs (320%–440%), but is likely CYP3A4 specific. Given the large and flexible active site of CYPs, the binding of several copies of a small molecule is possible, as has been shown for camphor in CYP101 (Follmer et al. 2018). Eleven different hits were shared only by CYP3A4 without ND and CYP3A4‐DMPC‐NDs, and these are more likely to be specific for CYP3A4 than the aforementioned hits for CYP3A4 in the absence of the membrane. These fragments similarly have a high average cLogP of ~2.1, showing significant reference binding for POPC NDs (−300%–0% occupancy) and no binding for DPhPC NDs (~30% occupancy). The DMPC NDs provided a more sensitive referencing system for SPR, leading to a larger number of detected hits than for the other NDs. In almost all instances of shared hits between CYP3A4 without ND and in DMPC NDs, the fragment occupancy was higher in NDs, indicating that fragment binding to CYP3A4 was directly facilitated through hydrophobic partitioning or otherwise promoted by the ND membrane. Another four hits were found for CYP3A4 inside all three types of NDs and not for CYP3A4 without ND. Importantly, two of these fragments did not bind significantly to the empty ND, and therefore, binding was measured specifically for CYP3A4. The other two fragments also bound to the empty ND model membrane but displayed a higher occupancy to CYP3A4 NDs comprising the same lipids. Additionally, two hits were shared only by CYP3A4 in DPhPC and DMPC NDs. The two fragments that bound to all of the immobilized targets are the best hit candidates. These fragments potentially coordinate to the heme iron in the active site of CYP3A4 via their aromatic nitrogen atoms. Example chemical structures of representative fragment hits are shown in Table S4.

## 3 Discussion

The sensitivity of SPR depends on the mass ratio between the analyte and the immobilized target, and their respective refractive index increments. Thus, for NDs, which are large in comparison (~150–200 kDa), high surface immobilization levels are required to obtain a sufficient signal‐to‐noise ratio for the screening of small fragments (< 300 Da). For this reason, the high‐affinity poly‐Ni‐NTA sensor chip (NiHC), which supports a high surface density of NDs, was used. However, leaching of CYP3A4 NDs from the surface was observed, and while the SPR responses could be normalized using fluconazole control injections throughout the screens, a stable surface immobilization is preferred. More stable surface chemistries for NDs include the use of high‐affinity non‐covalent interactions, such as biotin‐streptavidin (Hutsell et al. 2010), recently developed Nanodisc‐specific anti‐MSP antibodies (Nakagawa et al. 2023), or covalent capture coupling of the MSP to the chip surface (Kimple et al. 2010).

In this study, NDs comprising a single lipid type were used, which is a simplification of the physiological situation. MPs can have very specific membrane requirements for fold and function (Bogdanov et al. 2008), or their activity can be directly regulated by the presence of lipid cofactors (Cecchetti et al. 2021; Huang et al. 2022; Dijkman and Watts 2015). Model membranes should be able to solubilize the hydrophobic transmembrane regions of the MP (avoiding hydrophobic mismatch), while also permitting the outer‐ and/or inner‐membrane domains to protrude into the aqueous environment if necessary. All these issues need to be considered for each particular target, especially if the full activity of the target is relevant for the drug screen.

GPCRs represent the largest class of transmembrane receptors and hold significant interest for therapeutic modulation by small molecule drugs. However, they are recognized as unstable and challenging to purify to high homogeneity, and generally require thermal stabilization through mutation (StaRs) (Magnani et al. 2008; Robertson et al. 2011; Serrano‐Vega et al. 2008; Shibata et al. 2009). As a result of these stabilizing point mutations, StaRs become confined to adopting either an agonist or inverse agonist/antagonist conformation.

Reconstitution of GPCRs in NDs has demonstrated to improve stability (Bocquet et al. 2015), enabling structural studies by NMR (Kofuku et al. 2014; Guo et al. 2025) and CryoEM (Zhang et al. 2021), as well as biophysical interaction studies by microscale thermophoresis (MST) (Dijkman and Watts 2015) and SPR (Bocquet et al. 2015). Moreover, the native‐like ND lipid environment supports GPCRs in adopting biologically relevant conformations (Bocquet et al. 2015; Kofuku et al. 2014; Zhang et al. 2021; Whorton et al. 2008). The ND membrane also represents a more comprehensive model, as small hydrophobic or amphiphilic molecules are likely to traverse to hydrophobic sites of the receptor after membrane partitioning. Consequently, it is proposed that screening fragments or small molecules against GPCRs in NDs will identify more native conformation‐specific binders and result in better clinically translatable activities compared to screening GPCRs solubilized in detergent micelles.

The selection of an ideal ND lipid composition for the screening of other MPs such as GPCRs is difficult to generalize based on this study alone. Stricter selection criteria for membrane lipids may be imposed by flexible transmembrane proteins such as GPCRs, potentially hampering ND reconstitution yield or protein activity. ND assembly necessitates complete equilibration and removal of detergent above the main lipid phase transition temperature, complicating the reconstitution of unstable MPs in membranes composed solely of fully saturated linear acyl lipids such as DMPC (*T*_m_ = 24°C (Lewis et al. 2005)) and DPPC (*T*_m_ = 41°C (Lewis et al. 1996; Chen et al. 2018)). The incorporation of CYP3A4 in DMPC was feasible, but the ND reconstitution yield was much lower than when POPC or DPhPC was used. DPhPC NDs provided a good referencing system for SPR and are an attractive alternative to unsaturated acyl chain lipids for the reconstitution of unstable MPs.

The differences in screening effectiveness observed for CY3A4 highlight the importance of tailoring lipid composition to improve screening outcomes. Therefore, we recommend preliminary testing of different ND lipid compositions for each MP target, separately. Previous studies have demonstrated the use of various lipid mixtures for reconstituting GPCRs into NDs (Lavington and Watts 2020), which offer a valuable starting point for SPR screening of GPCRs in lipid NDs.

## 4 Conclusion

We have explored fragment screening of a prototypical MP, CYP3A4, incorporated into NDs comprising different phospholipids. Binding of small molecules and fragments to the empty ND model membrane was observed, correlating with analyte hydrophobicity. Extensive binding to the empty ND reference undermines the interpretation of analyte binding to the MP by SPR. Empty NDs prepared with the saturated, short‐chain acyl lipid DMPC (14:0) displayed significantly lower drug and fragment binding than the other lipids, in line with membrane partitioning data (Frallicciardi et al. 2022). Interestingly, we found clear differences in drug and fragment binding between POPC, DMPC, and DPhPC NDs. The latter two represent a better SPR referencing system than POPC NDs. CYP3A4‐DMPC‐NDs showed the highest fragment binding occupancy, and consequently the highest number of hits was detected using this ND. Furthermore, fewer binders were rejected based on atypical behavior for CYP3A4 NDs than for a non‐native oligomer of CYP3A4 without ND, suggesting that the binding to CYP3A4 was more specific in a membrane environment. Differences in the ligand access and egress channels of CYP3A4, in the presence or absence of the membrane, have been proposed based on molecular modelling (Lonsdale et al. 2014). The ND membrane was also shown to affect the cooperative binding of the small hydrophobic molecule testosterone (Baas et al. 2004). In line with these earlier works, certain fragments show higher binding occupancy to CYP3A4 in the presence of the ND membrane, or were exclusively detected as a hit for CYP3A4 inside NDs. An advantage of FBDD is the low molecular complexity of fragments, offering a good starting point for sampling potentially difficult‐to‐reach interfaces of MPs, while carrying a comparatively smaller hydrophobic baggage. That makes fragment screening a compelling method for MPs. With this study, it was demonstrated that the choice of the ND lipid can make a crucial difference in the outcome of a screen.

## Conflicts of Interest

The project was funded by the Dutch Research Council with financial contributions from Batavia Biosciences B.V. and ZoBio B.V.

## Supporting information


Data S1.

## Data Availability

The data that support the findings of this study are openly available in Zenodo at https://doi.org/10.5281/zenodo.14721783.
