# Supplementary material for: The Nature of Nanodisc Lipids Influences Fragment‐Based Drug Discovery Results
Source: Chem Biol Drug Des. 2025 Mar 14;105(3):e70080. doi: 10.1111/cbdd.70080 (PMC11909325; doi:10.1111/cbdd.70080)
Supplement: Supplementary file 1 — Data S1. [file CBDD-105-e70080-s001.docx]

**The nature of Nanodisc lipids influences fragment-based drug discovery results Supporting information**

Tim G. J. Knetsch^1^, Henri van Son^2^, Masakazu Kobayashi^2^, Marcellus Ubbink^1^*

^1^ Leiden Institute of Chemistry, Leiden University, Einsteinweg 55, 2333 CC Leiden, The Netherlands

^2^ ZoBio B.V., J.H. Oortweg 19, 2333 CH Leiden, The Netherlands

* Corresponding author, email: [m.ubbink@chem.leidenuniv.nl](mailto:m.ubbink@chem.leidenuniv.nl)


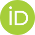
TK, 0000-0002-9575-5195; MU, 0000-0002-2615-6914; HS, 0000-0001-6206-2061

**Table of contents**

[Supplementary materials & methods 2](#_Toc174101115)

[Nanodisc assembly procedures 2](#_Toc174101116)

[Titration of fluconazole 2](#_Toc174101117)

[Sensor chip surface preparations 2](#_Toc174101118)

[SPR analysis of tool compounds 3](#_Toc174101119)

[SPR fragment screening 4](#_Toc174101120)

[Graphing and data analysis 4](#_Toc174101121)

[Supplementary figures 5](#_Toc174101122)

[Supplementary tables 8](#_Toc174101123)

[References 11](#_Toc174101124)

# Supplementary materials & methods

## Nanodisc assembly procedures

Dried lipids were solubilized by addition sodium cholate detergent until the solution became completely transparent. A 5-fold excess of NDs over CYP3A4 was used with the same lipid-to-MSP1D1 ratio as for empty NDs. The reconstitution mixtures were prepared at a final concentration of 5 mM lipid and 20 mM cholate and left to equilibrate near the main phase transition temperature of the lipids for 1 h, DPhPC-NDs were equilibrated and assembled at 4 °C. Detergent was removed by 48 h dialysis, and NDs were purified on a Superdex 200™ increase, G10/300 column (Cytiva). Absolute Mw of empty and CYP3A4-NDs were measured using a SEC-MALS system comprising a miniDAWN® TREOS®, DynaPro®, Optilab differential refractometer (Wyatt technology) and 1260 Infinity II multiple wavelength absorbance detector (Agilent) as described previously for empty NDs^1^, and CYP3A4 NDs^2^.

## Titration of fluconazole

Fluconazole titrations for 1 µM of CYP3A4 in solution or in NDs were conducted at 20 °C, in 0.1 M sodium phosphate (NaPi) pH 7.4, containing 0.1 M KCl and 20% glycerol, on a Cary 60 UV-Vis Spectrophotometer (Agilent). Fluconazole was dissolved in DMSO for titration and the final DMSO concentration was < 1% (v/v). The apparent dissociation constant (*K*_D,app_) was determined from the absorbance change (difference spectra, peak-to-trough at ΔAbs_408-428nm_) as function of the ligand concentration by fitting to a 1:1 binding model (eq. 1).

$\Delta Abs =\frac{{Abs}_{max} \cdot[L]}{\left. K_{D, app} + [L \right]}$ (1)

Where ∆*Abs* is the difference in absorbance, *Abs*_max_ is the maximal absorbance change, and [*L*] is the ligand concentration.

## Sensor chip surface preparations

Biosensor experiments were performed on a Biacore 1S+ (Cytiva, Uppsala, Sweden). All experiments were done at 10 °C to ensure stability of the immobilized targets; CYP3A4 oligomer without ND, empty NDs, and CYP3A4 NDs. Flow cells of the poly-Ni^2+^-NTA, NiHC1000M chip (Xantec) were conditioned by three cycles of a 5 min injection of 350 mM ethylenediaminetetraacetic acid (EDTA) pH 8.5, followed by a 2 min injection of 50 mM NaOH at 10 μL / min. After conditioning, the surface was equilibrated for 2 min in 0.1 M NaPi, pH 7.4, 0.1 M NaCl (capture buffer). Then, the surface was prepared by a 2 min injection of 5 mM NiCl_2_ at 10 µL / min, followed by a 5 min injection of capture buffer to equilibrate the surface.

CYP3A4 NDs were prepared using MSP1D1 without the his-tag, which was cleaved off using TEV protease. The immobilized targets were diluted to 2 μM in capture buffer, and captured by Ni^2+^-affinity using the his-tag on MSP1D1 for empty NDs and the his-tag on CYP3A4 for the other samples. CYP3A4 without ND was captured to ~4000 RU, allowing a theoretical maximal response (*R*_max_) of ~15 RU for a 200 Da Fragment. Empty and CYP3A4 NDs were captured to 4000 – 6500 RU, depending on the ND Mw determined by SEC-MALS, allowing a theoretical *R*_max_ of 5 RU for a 200 Da Fragment. Depending on the experimental setup (Fig. 4), the empty NDs were immobilized in an active flow cell or a reference flow cell for the CYP3A4 NDs comprising the same phospholipids, to study the interaction of compounds to the empty NDs or to CYP3A4 NDs respectively. The empty channel reference was activated in a comparable manner, excluding the injection of CYP3A4 or NDs. After capturing of the immobilized target, the sensor chip surface was thoroughly equilibrated by 60 start-up cycles with 60 s association and 60 s dissociation times.

## SPR analysis of tool compounds

The binding late response was calculated from the average response measured for 5 s, a few seconds before the termination of each injection cycle. The analysis of fragment binding to NDs was based on fragment percent occupancy to correct for deviations in immobilization levels and Mw of the immobilized targets and analytes. The binding late response was converted into percent occupancy for each fragment, using Eqs. 2 and 3.

$Theoretical R_{max} (RU)= \frac{{Rim}_{Target}}{{Mw}_{Target}} \times{Mw}_{Analyte}$ (2)

$Occupancy \left( \% \right)= \frac{Binding late response (RU)}{Theoretical Rmax(RU)} \times100$(3)

Where *Rim*_Target_ is the immobilization level of the target protein / ND, *Mw*_Target_ and *Mw*_Analyte_ are the molecular weights for immobilized target and analyte respectively. The binding late response was extracted for each fragment concentration and was fit to the Langmuir isotherm equation (Eq. 4) to determine the equilibrium dissociation constant (*K*_D_).

$R_{eq}=\frac{R_{max} \cdot c}{K_{D}+c}$ (4)

Where *R*_eq_ is the steady state response observed from the injected analyte at concentration *c* and *R*_max_ is the maximum response when the one binding site is fully saturated by the analyte. *k*_on_, *k*_off_ and *K*_D_ were calculated by fitting the double referenced sensorgrams to a 1:1 kinetic model using Biacore Insight Evaluation software (cytiva) (Eq. 5).

$\frac{dR}{dt}=k_{on}\cdot c\cdot R_{max}-R\left( k_{on}\cdot c+k_{off} \right)$ (5)

Where *dR* is the change in response unit, *dt* is the change in time, *c* is the concentration of injected analyte, *R*_max_ is the maximum response observed when all binding sites on the immobilized protein are saturated, and *R* is the response units induced by analyte binding^3^.

## SPR fragment screening

To correct for leeching of CYP3A4 NDs from the SPR chip surface, the fragment responses were normalized using control injections of fluconazole at 100 μM, for 60 s with a 90 s dissociation time, throughout the screening. The blank corrected binding late response of the fragments and fluconazole controls were first adjusted by dividing by the Mw_Analyte_. The response was plotted as response per 100 Da within the Biacore Insight Evaluation Software. Next, the response levels of the fragments were normalized by transforming the responses using a linear equation fitted to the control fluconazole injection points (Fig. S4).

For screen B1 (Fig. 4), fluconazole was titrated at 12.5, 25, 50 and 100 μM before, in the middle and at the end of the screen, to determine the affinity and surface activity of the immobilized CYP3A4. The fluconazole titration range was optimized for screen B2: from 0.7 μM to 100 μM, resulting in a better coverage (Table S3 and Fig. S6).

## Graphing and data analysis

Molecular graphics were rendered with UCSF ChimeraX, developed by the Resource for Biocomputing, Visualization, and Informatics at the University of California, San Francisco, with support from National Institutes of Health R01-GM129325 and the Office of Cyber Infrastructure and Computational Biology, National Institute of Allergy and Infectious Disease^42–44^. SPR data were blank corrected, corrected for Mw_Analyte_, and normalized for fluconazole injections, when applicable, using the Biacore Insight Evaluation software. Other graphing, fitting of data, and statistical analyses were performed using OriginPro (OriginLab).

# Supplementary figures

***Figure S1.*** Binding of tool compounds (1 µM) to empty NDs and CYP3A4 NDs, measured by SPR. Empty NDs comprise POPC, DPPC, DPhPC or DMPC lipids (top). Binding to CYP3A4 was measured without ND and inside POPC and DMPC NDs (bottom). Tool compound binding to the empty ND was mostly insignificant at 1 µM (occupancy < 30%, ~4 RU). Binding to CYP3A4 was observed for ketoconazole and clotrimazole. For CYP3A4-POPC-NDs, several negative sensorgrams were obtained, indicating higher relative binding of compounds to the empty POPC ND, immobilized in the reference channel. The bars indicate the occupancy (%) (left axis). Dots indicate cLogP (right axis) and the line is drawn to guide the eye.


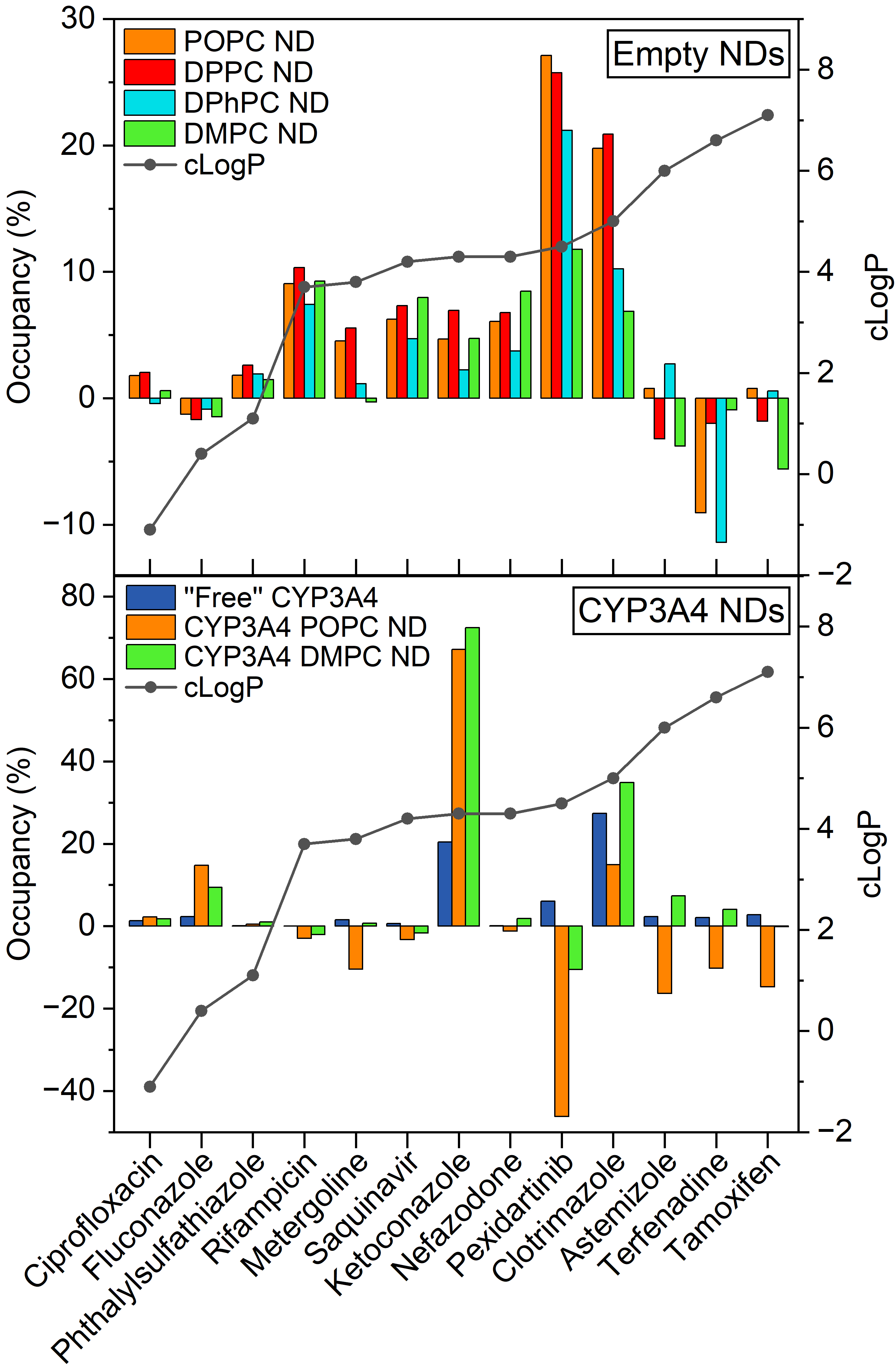

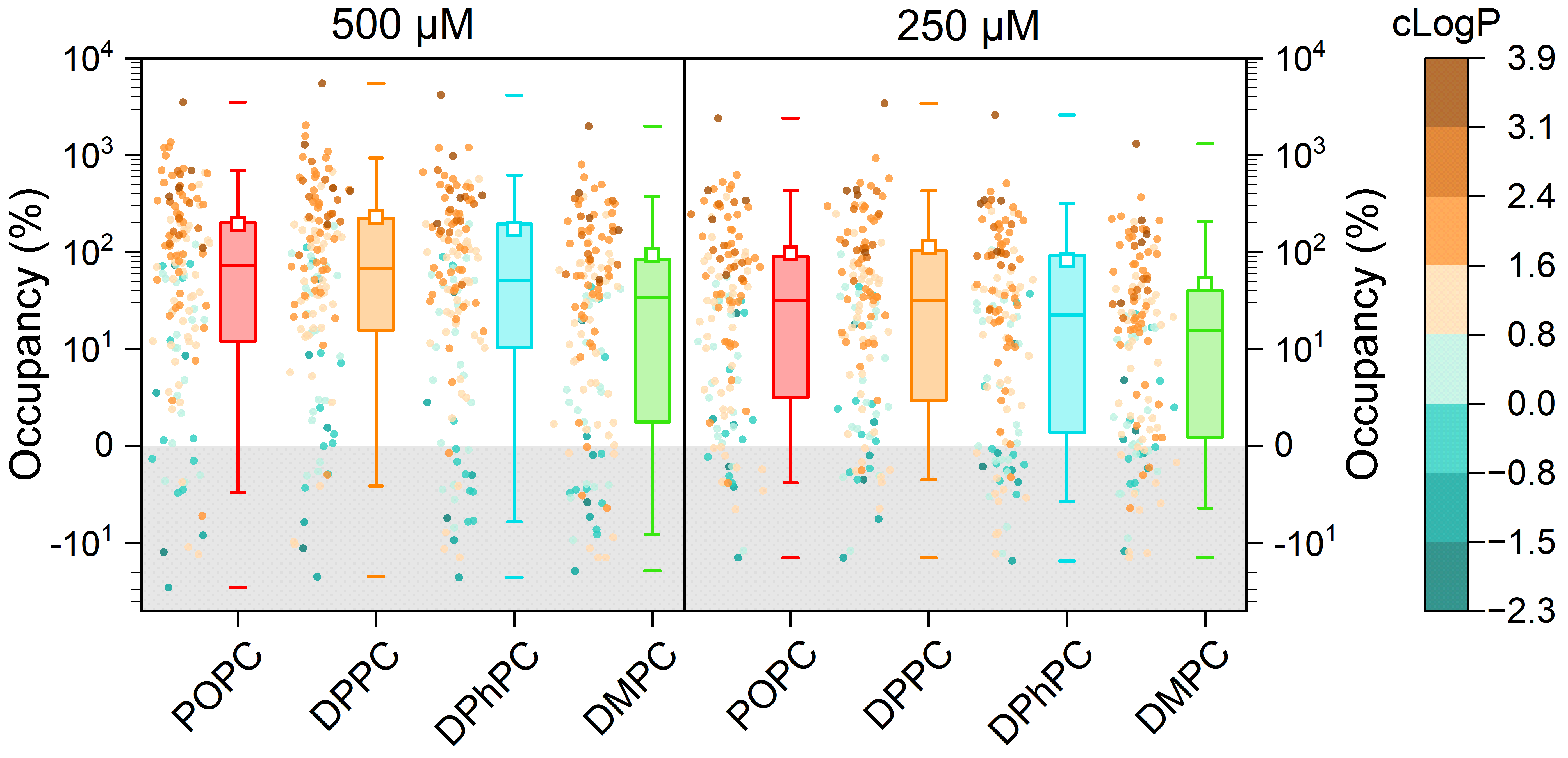


***Figure S2.*** Fragment binding (occupancy, %) to empty NDs at fragment concentrations of 500 µM (left) and 250 µM (right) in SPR. The boxes indicate the interquartile range (middle 50%), the median (line within the box), the average (open squares), the whiskers cover 5 – 95% of the data, and the outermost data points are indicated by the horizontal dashes. Binding data (dots) were colored according to fragment cLogP (scale on the right).


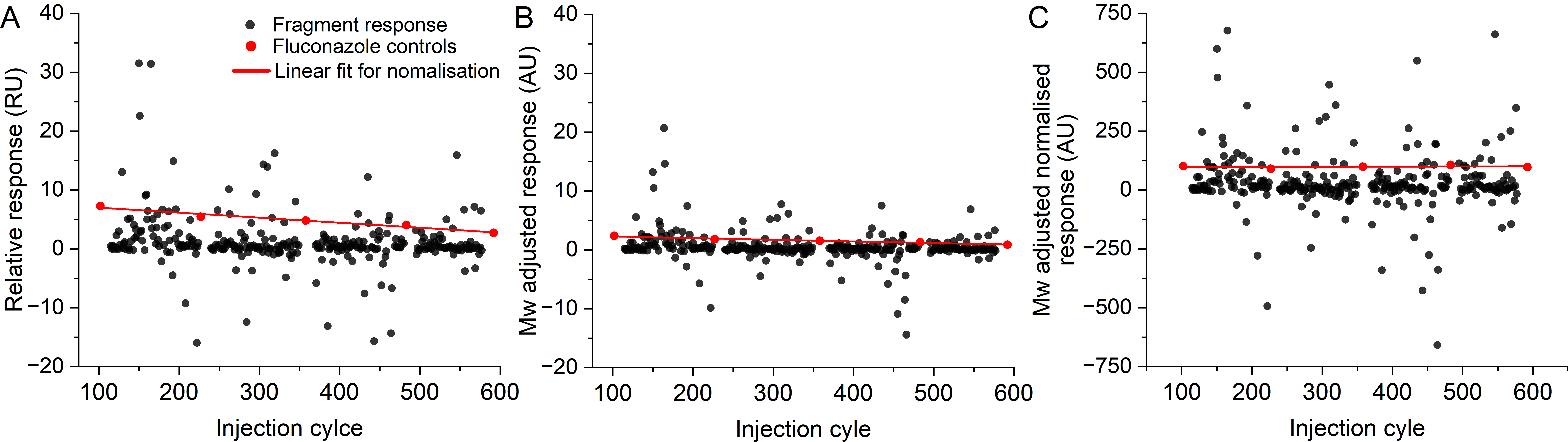


***Figure S4.*** Normalization of fragment binding response during the SPR screening of CYP3A4 in NDs. The fragment screen of CYP3A4 DMPC NDs is shown as an example. (**A**) The blank corrected binding late response is shown for the fragments (black dots) and 100 µM fluconazole control injections (red dots). The fluconazole injections were used to derive a linear equation for normalization (red line). (**B**) The blank corrected binding late response was first adjusted by dividing the response by the Mw_Analyte_ and the responses were plotted per 100 Da. (**C**) The linear fit was transformed to a horizontal line at 100 RU. The response of each fragment was transformed accordingly to their position relative to the linear fit used for normalization.


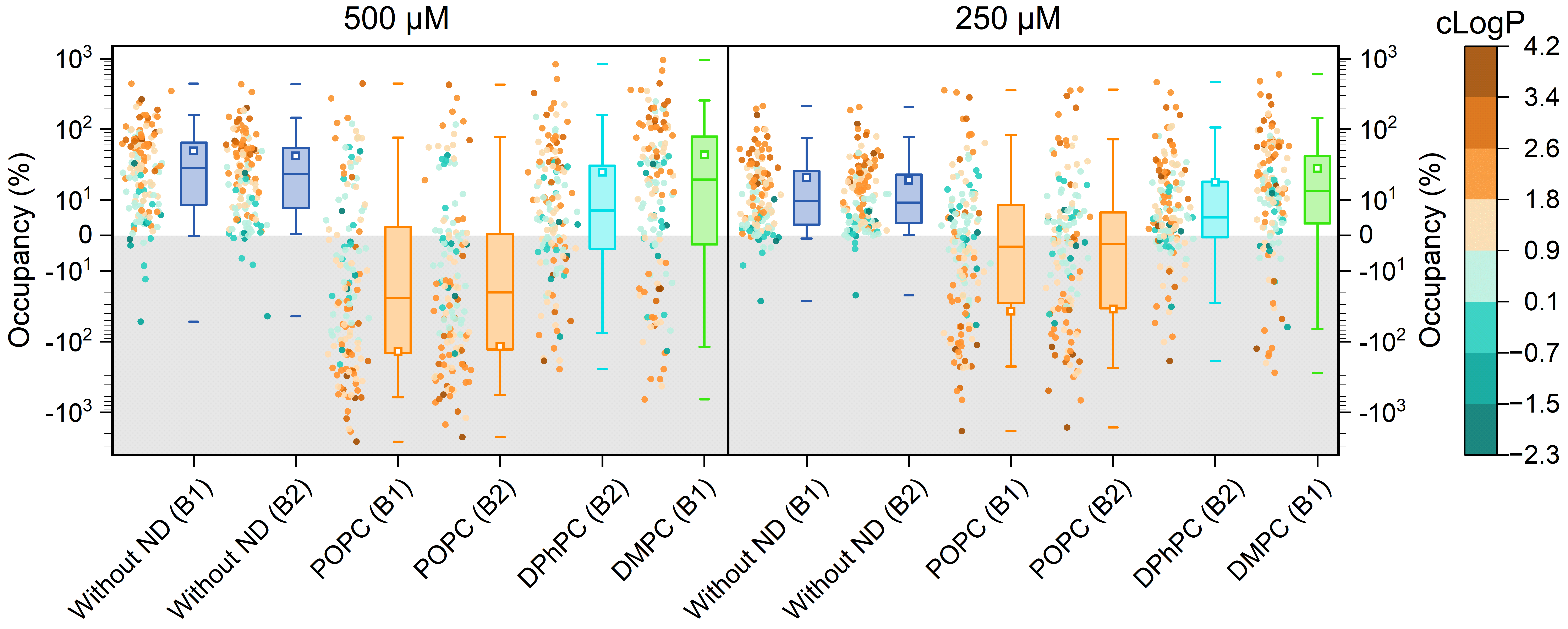


***Figure S3.*** Fragment binding (occupancy, %) at 250 and 500 µM to CYP3A4 without ND, and CYP3A4 NDs. The boxes indicate the interquartile range (middle 50%), the median (line within the box), the average (open squares), the whiskers cover 5 – 95% of the data, and the outermost data points are indicated by the horizontal dashes. Binding data (dots) were colored according to fragment cLogP (scale on the right). Note that for CYP3A4-POPC-NDs the binding to the reference (empty ND) is noticeably higher, resulting in a negative occupancy for the majority of the hydrophobic fragments. The responses for CYP3A4 NDs were normalized using fluconazole control injections (see method section).


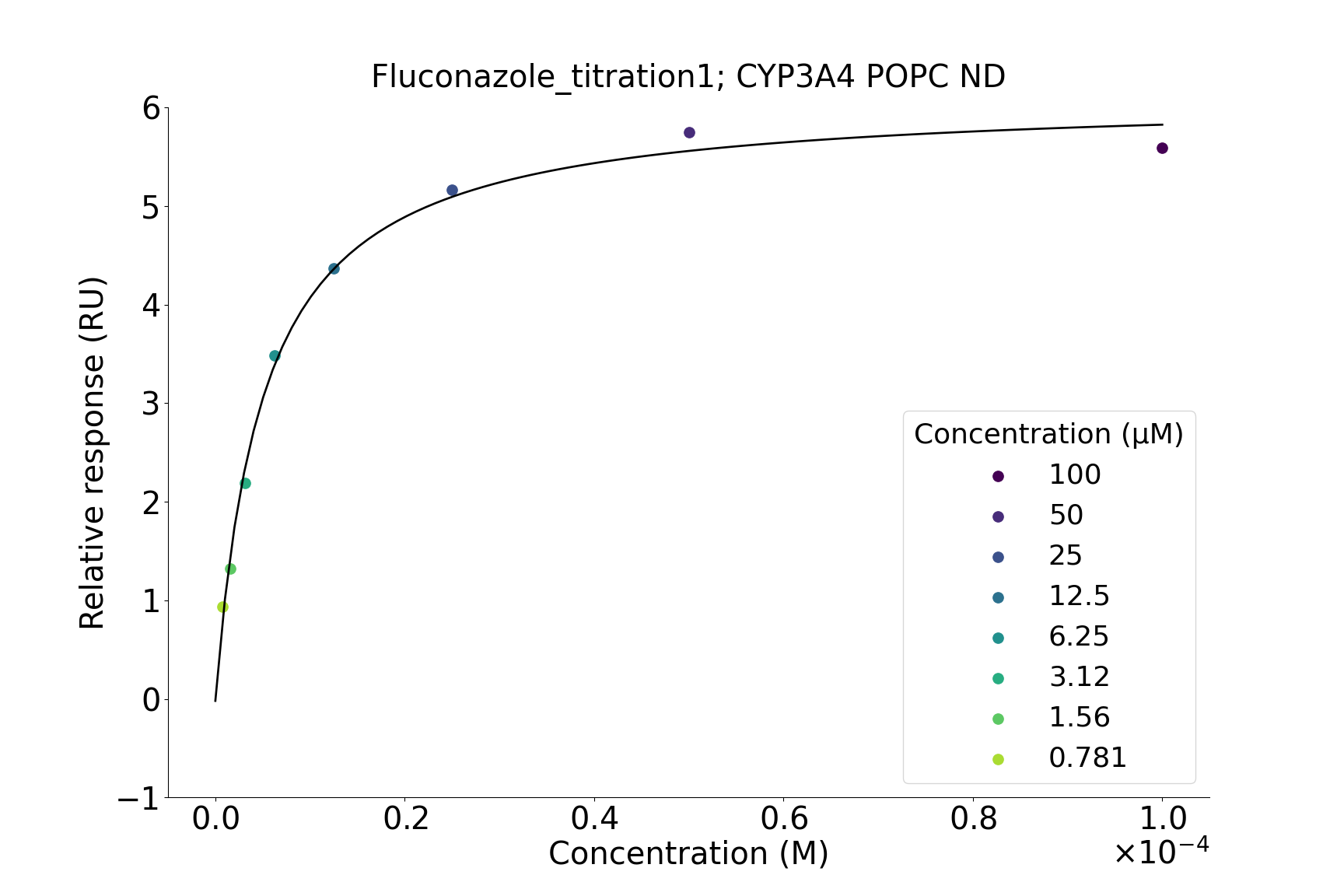


***Figure S6.*** Fluconazole binding to CYP3A4 in POPC NDs. Fluconazole was titrated onto immobilized CYP3A4 without or inside NDs. The SPR sensorgrams (left) for each concentration (colored lines) were fitted with a 1 : 1 kinetic binding model (black lines), to obtain the association and dissociation rate constants (*k*_on_ and *k*_off_), as well as the kinetic affinity constants (*K*_D, Kinetic_). The grey area represents the analyte binding late report points, of which the averages were plotted on the right. These average steady state data points were fitted using a steady state affinity model in the Biacore Insight Evaluation software, to determine the steady state affinity constant (*K*_D, Steady state_). Titrations were performed at 10 °C in 0.1 M KPi, pH 7.4, 0.15 M NaCl, 10 μM EDTA, 5% Glycerol, 1% DMSO. The kinetic parameters for fluconazole are shown in Table S3.


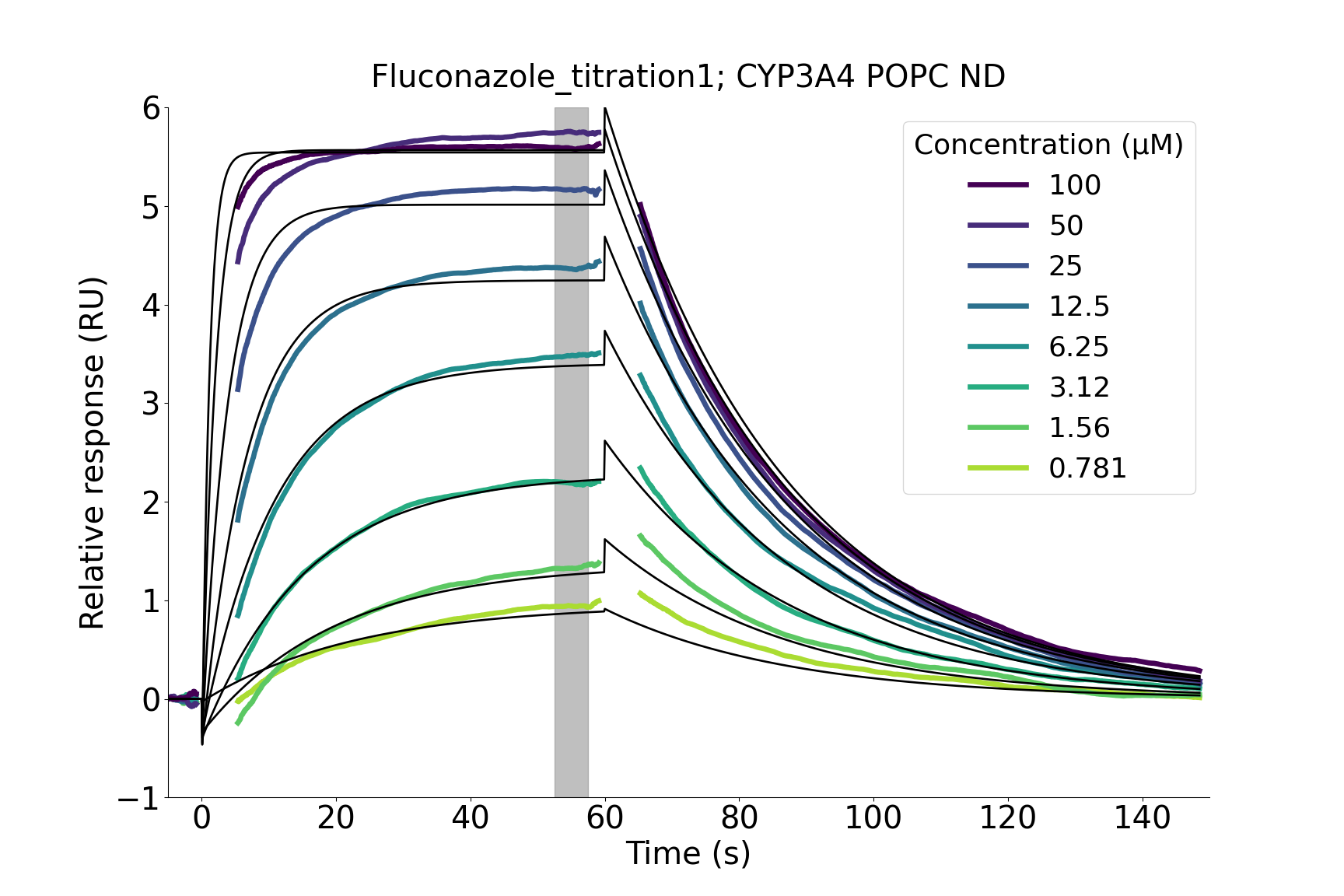


***Figure S5.*** Fluconazole binding to CYP3A4. Fluconazole was titrated against CYP3A4 in solution and in NDs, resulting in a type II spectral shift of the heme iron. The absolute and difference absorbance spectra are shown for CYP3A4 in POPC NDs, and were similar for all samples. The peak-to-trough absorbance difference spectra (ΔAbs_408-428 nm_) were used to determine the apparent *K*_D_ for fluconazole (eq. 1). The *K*_D,app_ values are 16, 14, 18 and 12 μM for CYP3A4 in solution, POPC-NDs, DPhPC-NDs and DMPC NDs, respectively. Titrations were performed at 20 °C, in presence of 1 µM of CYP3A4 (in NDs), 0.1 M NaPi pH 7.4, 0.1 M KCl, 20% glycerol. Fluconazole was dissolved in DMSO and titrated in small amounts, the final DMSO concentration was < 1% (v/v).


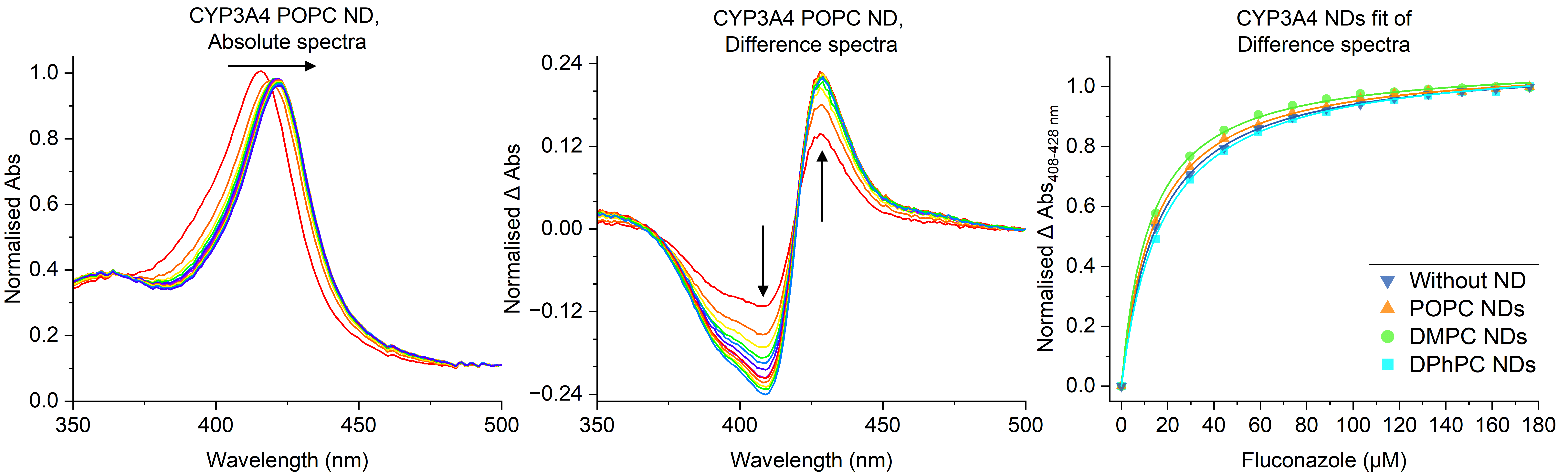


| ***Table S1.*** Physicochemical properties and ordering of the drug, tool compounds. | | | | | | | |
| --- | --- | --- | --- | --- | --- | --- | --- |
| Tool compound | Mw (Da) | cLogP^a^ | HBD^b^ | HBA^c^ | TPSA^d^ (Å^2^) | CAS# | Supplier |
| Ciprofloxacin | 331 | -1.1 | 2 | 6 | 75 | 85721-33-1 | MedChemExpress |
| Fluconazole | 306 | 0.4 | 1 | 7 | 82 | 86386-73-4 | MedChemExpress |
| Phthalylsulfathiazole | 403 | 1.1 | 3 | 8 | 126 | 85-73-4 | MedChemExpress |
| Rifampicin | 823 | 3.7 | 6 | 16 | 220 | 13292-46-1 | MedChemExpress |
| Metergoline | 404 | 3.8 | 1 | 5 | 47 | 17692-51-2 | Merck |
| Saquinavir | 671 | 4.2 | 6 | 11 | 167 | 127779-20-8 | MedChemExpress |
| Ketoconazole | 531 | 4.3 | 0 | 8 | 69 | 142128-57-2 | Merck |
| Nefazodone | 470 | 4.3 | 0 | 7 | 56 | 82752-99-6 | MedChemExpress |
| Pexidartinib | 418 | 4.5 | 2 | 5 | 67 | 1029044-16-3 | MedChemExpress |
| Clotrimazole | 345 | 5 | 0 | 2 | 18 | 23593-75-1 | Merck |
| Astemizole | 459 | 6 | 1 | 5 | 42 | 68844-77-9 | Merck |
| Terfenadine | 472 | 6.6 | 2 | 3 | 44 | 50679-08-8 | Merck |
| Tamoxifen | 372 | 7.1 | 0 | 2 | 13 | 10540-29-1 | MedChemExpress |
| ^a^ Computed logarithm of the octanol-water partition coefficient values were obtained from the Pubchem database  ^b^ H-bond donor count (HBD)  ^c^ H-bond acceptor count (HBA)  ^d^ Topological polar surface area (TPSA) | | | | | | | |

# Supplementary tables

| *Table S2.* ZoBio fragment screening library average physicochemical properties: Mw, cLogP, hydrogen bond donor count (HBD), hydrogen bond acceptor count (HBA), topological polar surface area (TPSA). | | | | | |
| --- | --- | --- | --- | --- | --- |
| Mw (Da) | cLogP | HBD | HBA | TPSA (Å^2^_­_) | Rotatable bonds |
| 204 | 1.3 | 1.0 | 2.4 | 47.8 | 1.9 |

| ***Table S3.*** Kinetic parameters determined from fluconazole binding titrations against CYP3A4 without and inside NDs, measured by SPR (as shown in Fig. S6.). | | | | | |
| --- | --- | --- | --- | --- | --- |
| Sample | Screen  (Fig 4) | *K*_D, Steady state_  x10^-6^ (M) | *K*_D, Kinetic_  x10^-6^ (M) | *k*_on_  x10^-3^ (M^-1^ s^-1^) | *k*_off_  x10^-3^ (s^-1^) |
| CYP3A4 without ND | B1^a^ | 31 | 7 | 2.6 | 1.9 |
| CYP3A4 without ND | B2 | 15 | 5 | 3.9 | 1.7 |
| CYP3A4-POPC-ND | B1^a^ | 6 | 5 | 8.8 | 4.8 |
| CYP3A4-POPC-ND | B2 | 4 | 5 | 8.8 | 3.7 |
| CYP3A4-DMPC-ND | B1^a^ | 6 | 4 | 9.7 | 4.2 |
| CYP3A4-DPhPC-ND | B2 | 12 | 6 | 6.4 | 3.6 |
| ^a^ During screen B1, the fluconazole titration range (12.5 to 100 μM) was insufficient. Thus, the derived kinetic parameters are less precise than for screen B2 where the titration range was increased (0.7 to 100 μM). | | | | | |

| ***Table S4*** Example fragment structures and binding occupancy (%) to empty and CYP3A4 NDs. Instances of fragment binding that were flagged for atypical behaviour or showed extensive binding to the sample in the reference channel are indicated by an asterisk. | | | | | | | | | | | | | | | |  |  |
| --- | --- | --- | --- | --- | --- | --- | --- | --- | --- | --- | --- | --- | --- | --- | --- | --- | --- |
| DPPC ND | POPC ND | | DPhPC ND | DMPC ND | | CYP3A4 without ND | CYP3A4-POPC-  ND | CYP3A4-DPhPC-ND | | CYP3A4-DMPC-  ND | | cLogP | | Structure | | |  |
| Amphiphilic fragments (low cLogP) that bind to empty NDs, and not to CYP3A4. | | | | | | | | | | | | | | | |  |  |
| 76 | 112 | 44 | | | 90 | 14 | -210* | | -12 | | -42 | | -0.2 | | 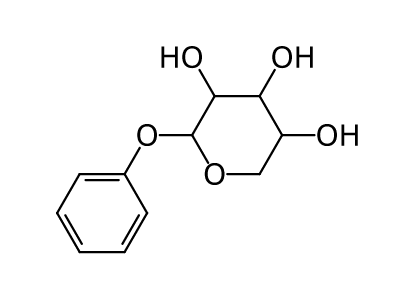 | | |
| 72 | 82 | 33 | | | 73 | 22 | -24 | | 12 | | 33 | | -0.6 | | 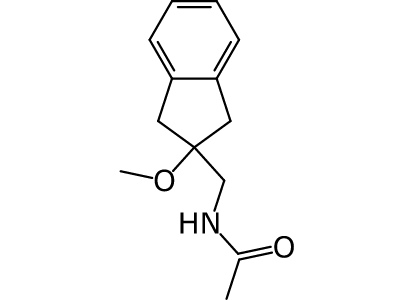 | | |
| Highest binder for all empty NDs. | | | | | | | | | | | | | | | |  |  |
| 3511 | 5503 | 1981 | | | 4187 | 266* | -2598* | | -187* | | -18* | | 3.7 | | 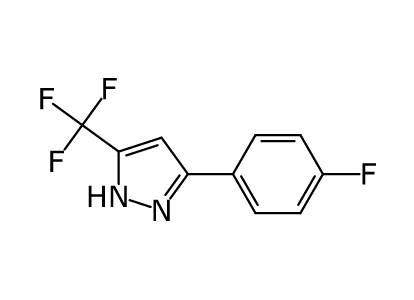 | | |
| Hits detected for CYP3A4 without ND only, but high chance of being aspecific. | | | | | | | | | | | | | | | |  |  |
| 631 | 861 | 704 | | | 340 | 71 | -479 | | -38 | | -27 | | 2.7 | | 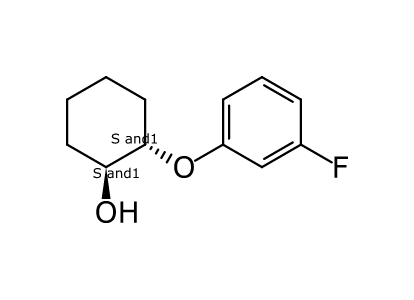 | | |
| 651 | 688 | 464 | | | 315 | 125 | -393* | | 49* | | 107* | | 2.1 | | 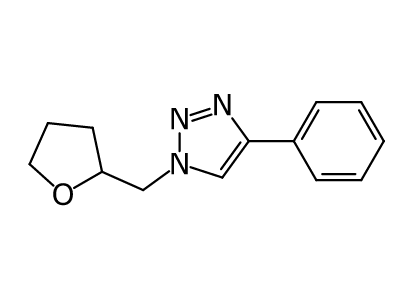 | | |
| Hits shared by CYP3A4 without ND and CYP3A4-DMPC-NDs, with higher binding to CYP3A4 inside DMPC NDs. | | | | | | | | | | | | | | | |  |  |
| 169 | 223 | 195 | | | 76 | 78 | -55 | | 33 | | 194 | | 0.8 | | 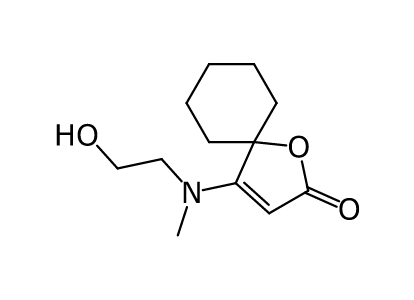 | | |
| 724 | 1391 | 1104 | | | 406 | 188 | -1226 | | -51 | | 197 | | 3.1 | | 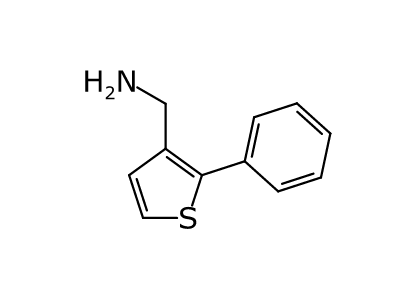 | | |
| Hits for CYP3A4 in NDs only. | | | | | | | | | | | | | | | |  |  |
| 35 | 41 | 41 | | | 20 | 29 | 94 | | 64 | | 105 | | 1 | | 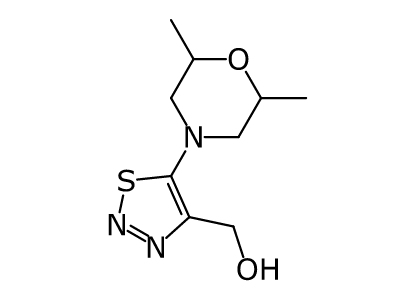 | | |
| 22 | 21 | 20 | | | 9 | 31 | 124 | | 79 | | 108 | | 1.9 | | 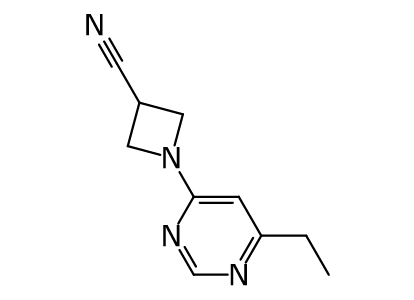 | | |
| Hit for all CYP3A4-containing samples, with and without ND. | | | | | | | | | | | | | | | |  |  |
| 169 | 146 | 122 | | | 78 | 97 | 56 | | 150 | | 181 | | 0.9 | | 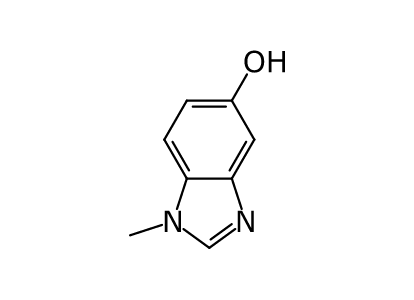 | | |
| 189 | 192 | 157 | | | 80 | 77 | 78 | | 79 | | 194 | | 1 | | 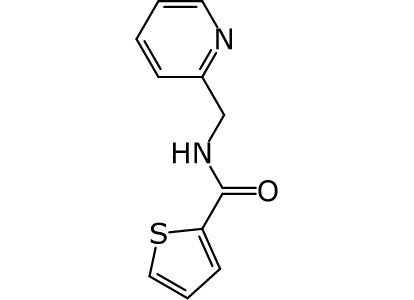 | | |
| Hit for CYP3A4, with a high stoichiometry for CYP3A4 NDs (was not counted as hit), fragment binding to CYP3A4 was likely increased in presence of the NDs, and this can be considered a hit for all samples. | | | | | | | | | | | | | | | |  |  |
| 149 | 93 | 46 | | | 49 | 144 | 444 | | 323 | | 447 | | 2.6 | | 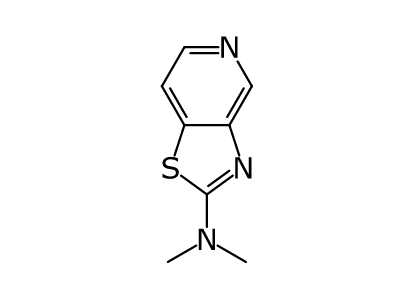 | | |
| Hit for all except for CYP3A4-POPC-ND, likely due to binding to the empty POPC ND reference. | | | | | | | | | | | | | | | |  |  |
| 198 | 345 | 233 | | | 97 | 112 | 38 | | 163 | | 262 | | 1.5 | | 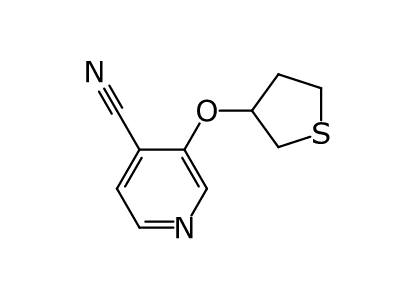 | | |
| 485 | 560 | 375 | | | 234 | 118 | -62 | | 104 | | 164 | | 2.4 | | 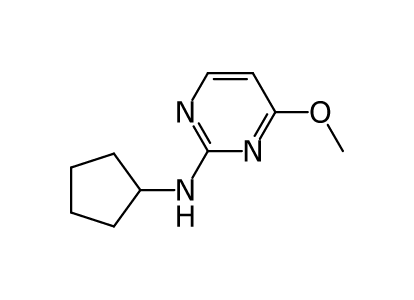 | | |
| Hits for CYP3A4 in DMPC and DPhPC NDs, *fragments were rejected as hits for CYP3A4 without ND. | | | | | | | | | | | | | | | |  |  |
| 111 | 194 | 197 | | | 52 | 68* | 21 | | 161 | | 251 | | 3.2 | | 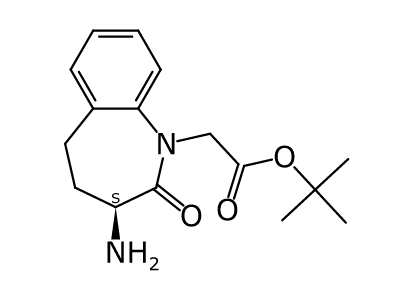 | | |
| 96 | 106 | 62 | | | 35 | 58* | -11 | | 72 | | 107 | | 1.8 | | 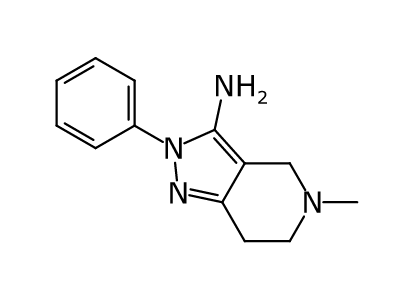 | | |
|  | | | | | | | | | | | | | | | |  |  |

# References

(1) Knetsch, T. G. J.; Ubbink, M. The Effect of Lipid Composition on the Thermal Stability of Nanodiscs. *Biochim. Biophys. Acta - Biomembr.* **2024**, *1866* (1), 184239. https://doi.org/10.1016/j.bbamem.2023.184239.

(2) Knetsch, T. G. J.; Ubbink, M. Lipid Composition Affects the Thermal Stability of Cytochrome P450 3A4 in Nanodiscs. *Biochim. Biophys. Acta - Biomembr.* **2024**, 184372. https://doi.org/10.1016/j.bbamem.2024.184372.

(3) GE Healthcare Life Science. Biacore TM Assay Handbook. **2012**, 1–78.
